# Dermis-retained breast dermo-glandular flap: a new surgical approach for granulomatous lobular mastitis

**DOI:** 10.3389/fsurg.2023.1187811

**Published:** 2023-06-16

**Authors:** Junying Huang, Tat-Hang Sin, Longzhu Nie, Yidong Zhou, Fan Zhang, Jia Ma, Xiaoguang Shi, Linlin Chen, Kunying Niu, Xiaohui Zhang, Qiang Sun, Hanyuan Huang

**Affiliations:** ^1^ Department of Breast Surgery, Peking Union Medical College Hospital, Chinese Academy of Medical Sciences & Peking Union Medical College, Beijing, China; ^2^ Department of Breast Surgery, Beijing Dangdai Hospital, Beijing, China

**Keywords:** granulomatous lobular mastitis (GLM), dermis-retained BDGF, breast, breast cancer, surgery

## Abstract

**Background:**

Granulomatous lobular mastitis (GLM) is characterized by nonspecific chronic inflammation concentrated in breast lobules. Surgical resection is one of the most common treatment options for GLM. On the basis of our previous use of Breast Dermo-Glandular Flap (BDGF), we designed a new surgical approach for GLM, especially for cases where the focus is close to the nipple. Here we describe this new treatment approach.

**Methods:**

In Peking Union Medical College Hospital (PUMCH) and Beijing Dangdai Hospital during January 2020—June 2021, we enrolled all 18 GLM patients who underwent surgery with the use of Dermis-Retained BDGF. All patients were women; most of the patients were 18–50 years old (88%); and the most common clinical manifestation of GLM was breast mass (60%). Then, we collected and analyzed data about the surgery and outcomes (drainage tubes moving time, relapse, patients’ shape satisfaction). We regarded GLM recurrence on the same side as relapse. If there was no complication and the patient's satisfaction was excellent or good, we rated the surgery as successful. We recorded the occurrence of all common postsurgical complications of the breast.

**Results:**

The debridement area was 3–5.5 (4.3 ± 0.7) cm; surgery time was 78–119 (95.6 ± 11.6) min; and mean debridement time (27.8 ± 8.9 min) was shorter than the time to obtain and transplant the flap (47.5 ± 12.9 min). Blood loss was less than 139 ml. As for bacterial culture, two patients had positive results, but they had no symptoms. No surgery-related complications happened. In terms of the outcomes, all of the drainage tubes were removed in less than 5 days, and only one patient experienced relapse after 1 year of surgery during the follow-up. The patients’ satisfaction with the breast shape was as follows: excellent (50%), good (22%), acceptable (22%), and poor (6%).

**Conclusion:**

For GLM patients refractory to conservative therapy or former unsatisfactory surgical management whose lesion is in the vicinity of the nipple and larger than 3 cm, Dermis-Retained BDGF is a suitable approach to fill the after-debridement defect below the nipple-areola and achieve a relatively satisfactory cosmetic outcome.

## Introduction

Granulomatous lobular mastitis (GLM) is characterized by nonspecific chronic inflammation concentrated in breast lobules, its etiology is still unknown (1). Although GLM is a benign disease, it is hard to cure. Recurrent attacks can damage the shape of the breast and reduce the quality of life. However, there is no clinical guideline to cure GLM, so the therapies are diversified, including antimicrobial therapy, corticosteroids, and surgery (2). A breast inflammatory lesion is one of the most common clinical manifestations of GLM, the main goal of breast surgeons is to remove the inflammatory focus fully in a small incision. In 2015, we introduced Breast Dermo-Glandular Flap (BDGF) for the treatment of periductal mastitis, which is a pedicle flap containing cutaneous, subcutaneous, and mammary gland tissue. It's suitable for large breast tissue defect patients after debridement surgery, which can be helpful to heal the incision and maintain breast shape; since then, we have done further research about the use of BDGF, and in 2020, we established a four-pattern surgical strategy with the use of BDGF (3, 4). Though BGDF had improved surgical success and better prognosis for GLM, there is still some progress to be made in large lesions closed to nipple-areolar area. During our clinical practice, we found removing lesions near the areolar can cause areolar collapse after surgery, along with a large incision for a good operative field to achieve complete excision Now, in order to avoid collapsing after removing a big focus near the areola, we have developed a new surgical approach on the basis of Dermis-Retained BDGF. In this research, we introduce Dermis-Retained BDGF, a new approach for the treatment of GLM.

## Material and methods

### Study design and patients

This is a descriptive retrospective study, we screened the case history for GLM cases at Peking Union Medical College Hospital (PUMCH) and Beijing Dangdai Hospital from January 2020 to June 2021. All the pathological diagnoses of patients are GLM, and all of them signed an informed consent form. In one of the following criteria, the patients underwent surgical treatment: (1) refractory to conservative therapy; (2) recurrence after abscess incision and drainage or fistula removal; and (3) recurrence after inflamed tissue removal. This study was approved by the Ethics Committee of PUMCH. All procedures were conducted in accordance with the ethical standards of the 1964 Helsinki declaration.

The collected data were divided into three categories: (1) Characteristics of the patients: age, body mass index, duration of GLM (counts from the onset of symptoms), clinical manifestation, history of treatment, other (smoking, drinking, trauma within 1 month, diabetes mellitus); (2) Surgery: debridement area, the longest diameter of the flap, surgery time, mean debridement time, time to obtain and transplant the flap, blood loss, bacterial culture, serious complications; (3) Outcomes: drainage tube removal time, relapse, patients’ shape satisfaction. Follow-up data were obtained from the outpatient clinic as regularly as possible in postoperative 12–30 months, and the median follow-up time was 19 months.

The inclusion criteria were as follows: (1) Pathological diagnosis is GLM, all the patients had undergone pathological examination by core needle biopsy before surgery; (2) Breast inflammatory lesions occupy less than one-fourth of the ipsilateral breast; (3) Complete medical history is available; (4) Single focus, or more lesions but in the same quadrant; (5) The nipple-areola area hadn't been influenced by inflammation; (6) No serious fundamental diseases could influence data; (7) The distance between the areola and the lesion is less than a half of the breast's radius. Exclusion criteria were as follows: (1) Breast inflammatory lesions occupy more than one-fourth of the ipsilateral breast; (2) Multiple quadrants involved; (3) Nipple-areola area was influenced; (4) Serious fundamental disease.

### Surgery: the use of dermis-retained BDGF

Dermis-Retained BDGF was based on the traditional T-shaped incision, plus an additional big crescent incision. The scar after the surgery mainly included the annulus areola incision (the edge of the crescent) and an additional radial incision out of the crescent. The incision was designed according to mammography and ultrasound findings. The entire inflammatory lesion needed to be removed include the involved skin and the inflammatory area. All operations were performed under general anesthesia.

**Figure 1:**
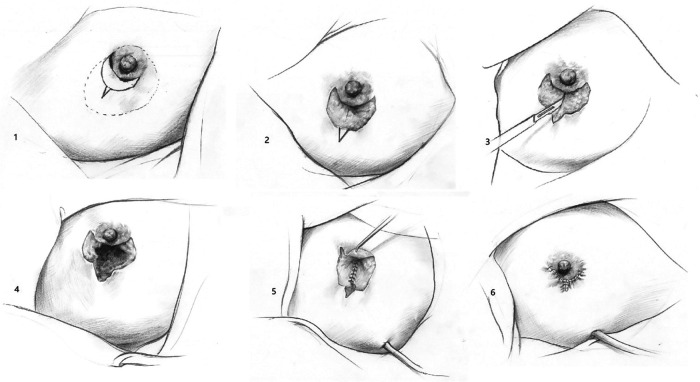
Surgical manuscript.

The operative steps were as follows (Figure 1):

(1) Modifying incision (full line) based on determining focal, size, range, and location (dotted line).
(2) In the crescent area, we remove the epidermis and reserve the dermis.
(3) T-shape entire-layers incision: cut the upper edge of the crescent fully, cut the dermo-glandular flap vertically and extended the incision to the outside of the lower edge of the crescent.
(4) Remove the inflammatory lesions completely.
(5) Retract the dermis reserved dermo-glandular flap to fill the defect under the nipple-areolar area.
(6) Modifying the flap to plastic suture. Close the crescent dermal area first, then sew the upper and lower edge, and radial incision finally.

Throughout the whole process, we need to clean out the inflammatory tissue under the nipple-areolar completely.

**Figure 2:**
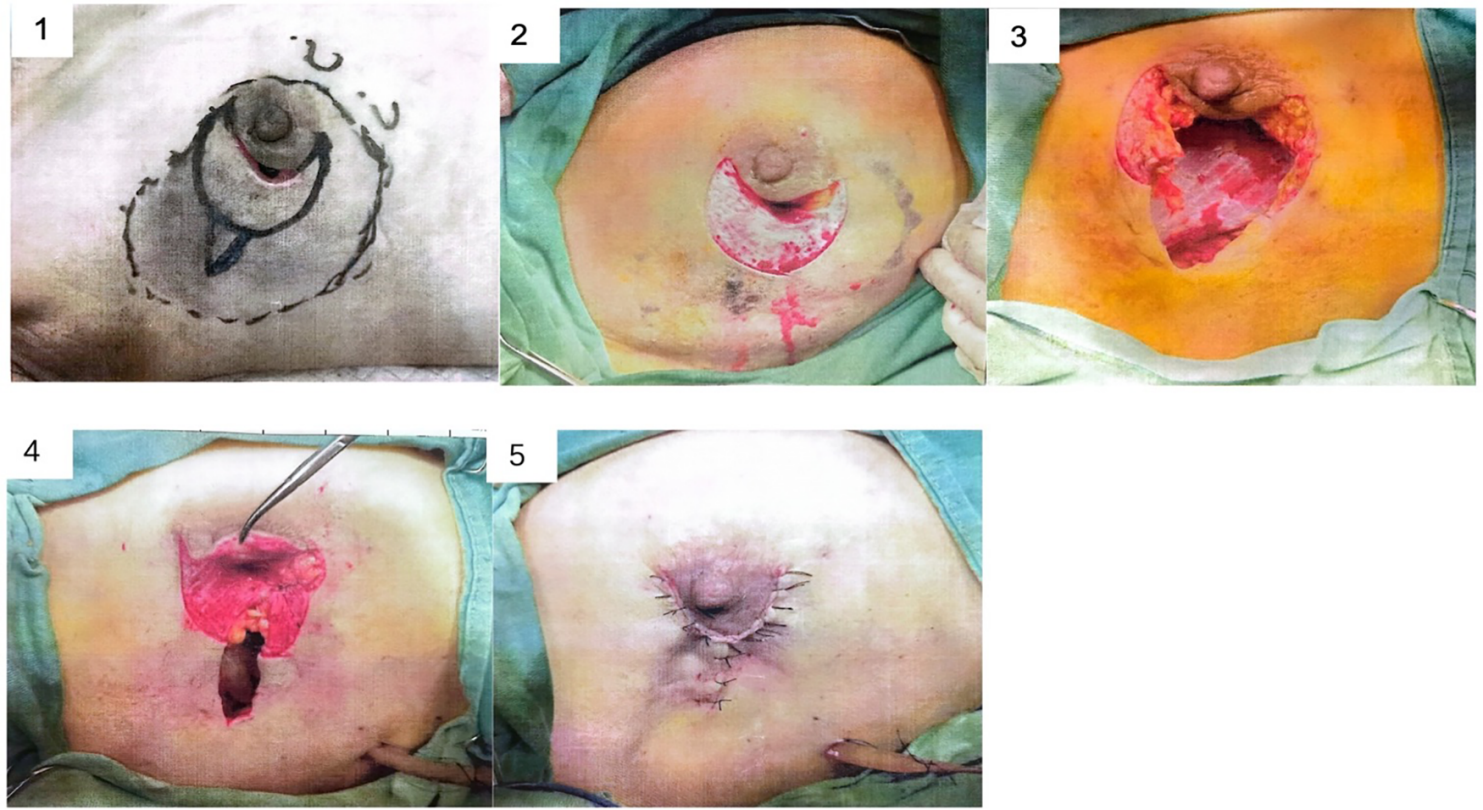
Real surgical photos.

Real surgical photos are shown in Figure 2.

### Evaluation standard

The information after hospitalization was obtained from the outpatient clinic during the follow-up of 12–30 months, median follow-up time was 19 months. The patients’ evaluation of the breast shape was based on the difference between the healthy side and the surgical side. We recognize the relapse of GLM in the same quadrant as recrudescence during the follow-up period. The complications of concern included subcutaneous fat liquefaction and necrosis, incision infection, skin flap ischemic necrosis, nipple–areola ischemic necrosis, ecchymoses, and hematoma.

### Statistical analysis

The data of the characteristics of the patients, surgery, and outcomes are presented as numbers and percentages. Statistical analysis was carried out using SPSS 16.0 (SPSS Inc., IL, USA).

## Results

### Characteristics of the patients

**Figure 3:**
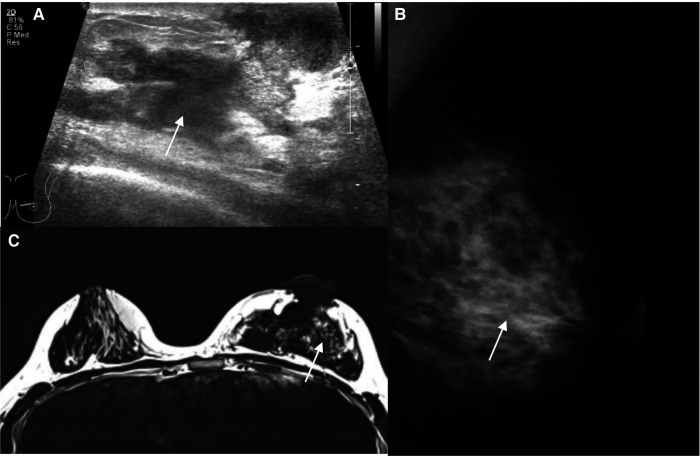
Image representation.

During our study period, 18 patients received the diagnosis of GLM. The clinical characteristics of the patients are shown in Table 1. Most of them were 18–35 years old (44%) or 35–50 years old (44%), and a big breast mass (>3 cm) was the main manifestation. The duration of GLM was usually more than 3 months, 39% > 6 months, 50% > 12 months. The lesion was carefully evaluated by mammography and ultrasound, and magnetic resonance imaging (MRI) was used in patients with multiple or extensive lesions. An example is shown in Figure 3, (A) Ultrasonography: GLM lesion of the left breast is a mixed area with hypoechoic and anechoic performance in ultrasonography, with an irregular margin; (B) Mammography: There is ill-defined diffusely asymmetric increased density below the nipple-areola area; (C) MRI: Inflamed tissues with high signal intensity near the areola area of the left breast is shown on T2 weighted MRI. More than half of the patients previously received conservative treatments such as antimicrobial therapy and corticosteroids.

**Table 1:** Characteristics of the patients with GLM.

Variables	Patients (*n* = 18)
Age (years), *n* (%)	
18–35	8 (44%)
35–50	8 (44%)
>50	2 (12%)
Body mass index (kg/m^2^), *n* (%)	
<18.5	0 (0%)
18.5–23.9	4 (22%)
24–26.9	8 (44%)
>27	6 (33%)
Duration of GLM (months), *n* (%)	
3–6	2 (11%)
7–12	7 (39%)
>12	9 (50%)
Clinical manifestation, *n* (%)	
Duct fistula	6 (33%)
Periareolar abscess	8 (44%)
Breast abscess (>3 cm)	12 (66%)
History of treatment, *n* (%)	
Corticosteroid therapy	11 (61%)
Antimicrobial therapy	10 (56%)
Surgical excision	9 (50%)
Surgical drainage	10 (56%)
Other, *n* (%)	
Smoking	2 (11%)
Drinking	3 (17%)
Trauma within 1 month	1 (6%)
Diabetes mellitus	1 (6%)

### Surgical data

The characteristics of surgery are shown in Table 2. Surgical time was 95.6 ± 11.6 min, and its major component included obtaining and transplanting Dermis-Retained BDGF (47.5 ± 12.9 min) and debridement time (27.8 ± 8.9 min). The debridement area is 4.3 ± 0.7 cm. The surgery was associated with minor blood loss, 116.3 ± 17.7 ml, and there were no serious complications during the follow-up period. Bacterial culture was positive in 2/18 patients (one *Staphylococcus aureus*; one *Staphylococcus epidermidis*).

**Table 2:** Characteristics of surgery.

Variables, *n* (%)	Minimum	Maximum	Mean ± SD
Debridement area	3.0 cm	5.5 cm	4.3 ± 0.7 cm
Surgery time	78.0 min	119.0 min	95.6 ± 11.6 min
Mean debridement time	16.0 min	46.0 min	27.8 ± 8.9 min
Time to obtain and transplant Dermis-Retained BDGF	29.0 min	70.0 min	47.5 ± 12.9 min
Blood loss	83.0 ml	139.0 ml	116.3 ± 17.7 ml
Bacterial culture	2 + (1 *Staphylococcus aureus*; 1 *Staphylococcus epidermidis*)		
Serious complications (which influence prognosis)	None		

### Outcomes after surgery

The patients’ satisfaction with the breast shape was obtained 1 year later. As shown in Table 3, all drainage tubes were removed within 5 days. Only one patient experienced a relapse in 1 year, the patient's new lesion appears on the inner top of the original lesion and it was successfully treated with a second operation. As for shape satisfaction, nine patients rated the shape as “excellent,” and only one patient rated the shape as “poor.”

**Table 3:** Outcomes after the new approach.

Variables	Patients (*n* = 18)
Drainage tube removal time (days), *n* (%)	
<3	11 (51%)
<5	18 (100%)
Recurrence (years), *n* (%)	
1	1 (6%)
Shape satisfaction, *n* (%)	
Excellent	9 (50%)
Good	4 (22%)
Acceptable	4 (22%)
Poor	1 (6%)

**Table:** 

RANK	DESCRIPTION (COMPARE THE AFFECTED SITE WITH THE HEALTHY SITE)
EXCELLENT	Affected site is almost the same as the healthy site
GOOD	There is kind of difference between the affected site and the healthy site
ACCEPTABLE	Less than one-fourth of the affected site is obviously different from the healthy site
POOR	More than one-fourth of the affected site is obviously different from the healthy site

## Discussion

GLM is one of the most common forms of non-lactation mastitis. It was first described by Going et al. in 1987 (5). GLM has region-specific characteristics. Namely, in developed countries, especially Turkey, the rate of GLM is higher than that in developing countries. Besides, the clinical presentation and treatment differ between countries (2). The specific cause of GLM is still unknown, so GLM is also known as idiopathic granulomatous lobular mastitis. According to existing literature, the suggested causes of GLM are infection and autoimmunity. Patients can be of any age but mostly between 30 and 40 years (6). Clinical manifestations can be divided into local and systemic manifestations. A single breast inflammatory lesion is the most common local presentation, along with inflammation, such as pain, swelling, redness, and warmth; systemic symptoms can also appear, such as aversion to cold; if the focus is hard to cure, a sinus tract or fistula can appear; if it damages the nipple–areolar area, it could lead to nipple collapse, discharge, and deformation (7). As for hormonal status, FSH/LH and PRL levels are associated with GLM recurrence (8). GLM has no specific clinical imaging findings and symptoms, but its pathology is distinct, so its definitive diagnosis can only be made through pathology. The histological feature is granulomatous inflammation concentrated in breast lobules, which contains numerous Langhans’ giant cells, epithelioid histiocytes, and neutrophil leukocytes (9).

Based on our data, the age of GLM patients was over 18 years, mostly between 18 and 50 years. The most common manifestation was a breast lesion (>3 cm). Since GLM does not have any specific clinical symptoms or clinical imaging findings, the definitive diagnosis can only be made through pathology.

There is no standard treatment for GLM. The existing methods include endocrine therapy, immunotherapy, traditional Chinese medical herbal treatment, anti-tuberculosis treatment, and surgical operation (10–12). For relapsing GLM or when the conservative treatment is not successful, surgery is recommended. Appropriate time of intervention is considered the key for a successful surgery; It has been suggested that surgery can be performed when the lesion is limited to one quadrant (13). The critical factor for reducing recurrence is to remove the lesion fully. For a big inflammation focus close to the areola, extended excision could result in vacuity within the areola, leading to its collapse after further sewing up. To solve the problem, we improved our surgical approach by designing Dermis-Retained BDGF, which can maintain breast shape satisfaction on the basis of clear removal of the incision.

We believe that Dermis-Retained BDGF is better for GLM with a big inflammatory lesion (>3 cm) and for a lesion close to the nipple–areolar area. Its biggest advantages are the support of a big view for surgical doctors and a good shape satisfaction for patients. First, the use of Dermis-Retained BDGF can prevent the collapse of the nipple–areolar area after removing the inflammatory lesion. Second, it can offer excellent support and blood supply for the nipple, which can avoid nipple collapse and necrosis. Third, Dermis-Retained BDGF and developed T-shaped incision reserve a nice operative field, also shorten the longitudinal incision by moving the Dermis-Retained part to the areolar area during sewing. Our new incision is suitable for filling up the vacuity after debriding a big lesion near the nipple. Patients whose lesion is near the boundary of the breast are not suitable for this incision, and those with a small inflammatory focus require a direct excision rather than a transplant flap. In the above conditions, there is relatively more tissue for the surgeon to remove and perform shaping. In some extreme conditions, for patients whose inflammation has destroyed the nipple–areolar area, it is not appropriate to keep the nipple–areola complex.

Since this research is designed as a single-arm pilot study for the certain lesions close to the nipple-areola area, together with the limited eligible patients’ numbers, we did not design a control group. Compared with our previous data about the primary BDGF surgical treatment of mastitis, this improved Dermis-Retained BDGF approach did not result in more bleeding or longer operative time, and there are no significant increases in both surgical complication and recurrence rate (3, 4).

The crucial factors for the use of Dermis-Retained BDGF incision are as follows: (1) The incision is centered on the inflammation, so we must evaluate the patient's medical imaging findings and feel the breast inflammatory lesion carefully. (2) We need to remove the lesion radically to prevent relapsing. (3) We wash the wound with sterile normal saline radically. (4) We indwell wound drain and keep negative pressure suction, and remove the drain when the drainage is <10 ml. (5) We wrap up the wound and keep pressing it. (6) We use antibiotics. However, our study has some limitations, including the lack of a control group of other treatment, and the short follow-up period.

## Conclusion

Surgery is an appropriate treatment for relapsing GLM or when conservative treatment is not successful. The key to a successful operation is to remove the lesion radically and choose an appropriate intervention time. In some cases, with a big inflammatory focus close to the nipple–areolar area, the improved T-shaped incision mentioned in this paper is a good choice. Our approach can ensure the curative effect and keep the beauty of the breast after the operation. Therefore, we think it is worth further promotion.

## Data Availability

The original contributions presented in the study are included in the article, further inquiries can be directed to the corresponding authors.
